# Effects of curcumin and/or coenzyme Q10 supplementation on metabolic control in subjects with metabolic syndrome: a randomized clinical trial

**DOI:** 10.1186/s12937-022-00816-7

**Published:** 2022-10-03

**Authors:** Abbas Ali Sangouni, Maryam Taghdir, Javad Mirahmadi, Mojtaba Sepandi, Karim Parastouei

**Affiliations:** 1 Health Research Center, Life Style Institute, Baqiyatallah University of Medical Sciences, Tehran, Iran; 2 Department of Nutrition, School of Public Health, Shahid Sadoughi University of Medical Sciences, Yazd, Iran; 3 Student Research Committee, Baqiyatallah University of Medical Sciences, Tehran, Iran

**Keywords:** Metabolic syndrome, Curcumin, Coenzyme Q10, Lipid profile, Blood pressure, Body composition

## Abstract

**Background:**

Metabolic syndrome (MetS) as a cluster of conditions including hyperlipidemia, hypertension, hyperglycemia, insulin resistance, and abdominal obesity is linked to cardiovascular diseases and type 2 diabetes. Evidence suggested that intake of curcumin and coenzyme Q10 may have therapeutic effects in the management of MetS.

**Aims:**

We investigated the effects of curcumin and/or coenzyme Q10 supplementation on metabolic syndrome components including systolic blood pressure (SBP), diastolic blood pressure (DBP), waist circumference (WC), triglyceride (TG), high density lipoprotein-cholesterol (HDL-c) and fasting plasma glucose (FPG) as primary outcomes, and total cholesterol (TC), low density lipoprotein-cholesterol (LDL-c) and body mass index (BMI) as secondary outcomes in subjects with MetS.

**Methods:**

In this 2 × 2 factorial, randomized, double-blinded, placebo-controlled study, 88 subjects with MetS were randomly assigned into four groups including curcumin plus placebo (CP), or coenzyme Q10 plus placebo (QP), or curcumin plus coenzyme Q10 (CQ), or double placebo (DP) for 12 weeks.

**Results:**

The CP group compared with the three other groups showed a significant reduction in HDL-c (*P* = 0.001), TG (*P* <  0.001), TC (*P* <  0.001), and LDL-c (*P* <  0.001). No significant differences were seen between the four groups in terms of SBP, DBP, FPG, WC, BMI and weight.

**Conclusion:**

Curcumin improved dyslipidemia, but had no effect on body composition, hypertension and glycemic control. Furthermore, coenzyme Q10 as well as the combination of curcumin and coenzyme Q10 showed no therapeutic effects in subjects with MetS. The trial was registered on 09/21/2018 at the Iranian clinical trials website (IRCT20180201038585N2), URL: https://www.irct.ir/trial/32518.

## Introduction

Metabolic syndrome (MetS) is a cluster of metabolic disorders such as hyperlipidemia, hypertension, hyperglycemia, insulin resistance, and abdominal obesity [1, 2]. MetS is associated with cardiovascular disease (CVD), type 2 diabetes mellitus (T2DM), and non-alcoholic fatty liver disease (NAFLD) [3–5]. The worldwide prevalence of MetS is increasing dramatically, and has become a major public health concern [1, 6]. Due to different diagnostic criteria like the National Cholesterol Education Program-Adult Treatment Panel III (NCEP-ATP III), the American Heart Association/National Heart, Lung, and Blood Institute (AHA/NHLBI), and the International Diabetes Federation (IDF), the exact prevalence of metabolic syndrome is unclear [4, 7, 8]. The global prevalence of MetS is between 14 and 32% [6]. A combination of genetic, metabolism and environmental factors is involved in the etiology of MetS [2, 9]. Insulin resistance and obesity play an important role in the pathogenesis of MetS [2, 10]. In addition to pharmacological management, lifestyle changes such as adherence to healthy dietary patterns, weight loss and physical activity are the important strategies in the management of MetS [11, 12].

Curcumin is a phytochemical and an active compound of turmeric (*Curcuma longa L.*) [13]. Evidence has shown some beneficial effects of curcumin on obesity, hypertension, dyslipidemia and glycemic control [14–17]. However, there is only one study (with limitations like short intervention duration) examining the effects of curcumin on the components of the MetS in subjects with MetS [18].

On the other hand, coenzyme Q10 as an antioxidant plays a critical role in scavenging active oxygen species and reducing lipid peroxidation, cellular signals, and energy production in mitochondria [19, 20]. Moreover, coenzyme Q10 increases insulin sensitivity and improves glycemic control [21]. Clinical findings regarding the effects of coenzyme Q10 on dyslipidemia, hypertension, and obesity are conflicting. Furthermore, some trials investigated the effect of coenzyme Q10 on the MetS components [22–26], but the results were not integrated.

In the present study we aimed to investigate the effects of curcumin and/or Coenzyme Q10 supplementation on metabolic syndrome components including diastolic blood pressure (DBP), systolic blood pressure (SBP), waist circumference (WC), triglyceride (TG), high density lipoprotein-cholesterol (HDL-c) and fasting plasma glucose (FPG) as primary outcomes, and total cholesterol (TC), low density lipoprotein-cholesterol (LDL-c), weight and body mass index (BMI) as secondary outcomes in subjects with MetS. We hypothesized that curcumin and/or coenzyme Q10 supplementation may improve cardiometabolic outcomes in subjects with MetS.

## Methods

### Recruitment and eligibility screening

Two hundred volunteer with MetS who referred to the department of Health, Assistance and Treatment in Tehran, affiliated with Baqiyatallah University of Medical Sciences in Tehran, Iran were identified and screened. The MetS defined by IDF is a clustering of elevated WC (≥94 cm for men and ≥ 80 cm for women) in conjunction of two or more of the following disorders: 1- elevated TG ≥150 mg/dL (1.7 mmol/L) (or drug treatment); 2- reduced HDL-c < 40 mg/dL (1.0 mmol/L) for men, and < 50 mg/dL (1.3 mmol/L) for women (or drug treatment); 3- elevated SBP ≥130 and/or DBP ≥85 mmHg (or antihypertensive drug treatment); and 4- elevated FPG ≥100 mg/dL (or drug treatment) [27]. In order to identify subjects with MetS, variables of MetS including WC, TG, HDL-c, SBP, DBP and FPG were measured. Subjects with elevated WC (≥94 cm for men and ≥ 80 cm for women) were referred to the laboratory to measure other factors of MetS. The inclusion criteria included subjects with MetS diagnosed by IDF guideline and aged ≥18 y. The exclusion criteria included history of cancers, cardiovascular disease, stroke, kidney diseases, and viral hepatitis, taking blood pressure lowering, blood lipid lowering, and blood glucose lowering medications, and unwillingness to continue the study. Finally, 88 subjects were included.

### Study design

This study was a 2 × 2 factorial, randomized, double-blinded, placebo-controlled study was conducted for 12 weeks. According to studies that investigated the effect of curcumin [28, 29] and coenzyme Q10 [22, 30], the duration of this study was determined to be 12 weeks. After explanation about the risks and benefits of the trial by investigator, an informed written consent approved by the ethics committee in Baqiyatallah University of Medical Sciences (IR.Bmsu.rec.1397.449) was signed by participants. In addition, participants could leave the trial at any time for any reason. Registration of the trial protocol was performed on 09/21/2018 at the Iranian clinical trials website (IRCT20180201038585N2), URL: https://www.irct.ir/trial/32518. At the beginning of the trial, subjects were randomly assigned into four groups including curcumin (daily one capsule contained 200 mg curcumin powder) plus placebo (matching for coenzyme Q10) group (CP), or coenzyme Q10 (daily one capsule contained 60 mg coenzyme Q10) plus placebo (matching for curcumin) group (QP), or curcumin (daily one capsule contained 200 mg curcumin powder) plus coenzyme Q10 (daily one capsule contained 60 mg coenzyme Q10) group (CQ), or either double placebo (daily two capsules contained cellulose acetate) group (DP). Simple (unrestricted) randomization was performed using random allocation software [31] and numbers from 1 to 88. The randomized allocation and assignment of participants into intervention groups was performed by a trained person who was not involved in the trial. Supplement boxes were labeled as A or B or C by a person who was not involved in the trial. Subjects and all investigators were blinded to the intervention assignment until the end of the study.

### Intervention

Every 4 weeks, capsules were given to participants. Participants consumed two capsules before lunch. Curcumin, coenzyme Q10 and placebo capsules were produced in Karen Pharmaceutical Co., Yazd, Iran. The appearance of curcumin, coenzyme Q10 and placebo was similar. The participants were advised to follow common healthy dietary recommendations (higher intake of fruits and vegetables, whole grains, legumes and fish, and lower intake of red and processed meats, full-fat dairy products, saturated fatty acids, refined sugars and salt) during follow-up. Every 4 weeks, the participants were asked to deliver empty boxes of capsules to the investigator. Consuming less than 80% of prescribed capsules was considered as poor compliance.

### Dietary intakes and physical activity evaluation

Evaluation of the energy and macronutrients intakes was performed by a 24-h recall questionnaire for 3 days (1 weekend day and 2 nonconsecutive weekdays) at baseline and after intervention. A metabolic equivalent of task (MET) questionnaire [32] was used for assessment of physical activity at baseline and after intervention. The validity of the MET questionnaire has been confirmed [33].

### Anthropometric evaluations

Height, weight and WC were measured at the baseline and the end of study. A stadiometer was used to measure height of participants, while the participants were in standing position without shoes. Weight was measured by a digital Seca scale (Seca, Germany) with an accuracy of 100 g. To measure weight, the participants were with light clothes and without shoes. The WC was measured at the middle point between the last noticeable rib and the top of iliac crest using a measuring tape with an accuracy of 0.5 cm. BMI was calculated by the following formula: weight (kg)/height (m)^2^.

### Blood pressure measurement

Measuring SBP and DBP was performed in the nondominant arm after 10 min of rest at baseline and after intervention. Using a valid mercury sphygmomanometer device (MicrolifeBP AG1–10), the participant’s blood pressure sitting at the suitable position (subject’s arm at the same level with the heart) was measured 3 times. The mean of the 3 measurements were used in the analyses.

### Laboratory assessments

To measure serum FPG, TG and HDL-c concentrations at baseline and after intervention, blood was drawn after 12 hours fasting and was centrifuged for 10 minutes at a speed of 3600 rpm. Serum samples were evenly poured into the microtubes immediately frozen at − 70 °C. Serum FPG, TG and HDL-c were measured by routine enzymatic assays with commercial kit (Pars Azmoon, Iran) using an autoanalyzer (AVIDA 1800 chemistry system; Siemens, United Kingdom). Measurements were done under the standard methods in laboratory of Nutrition Department.

### Statistical analysis

Sample size was calculated based on the study of Panahi et al. [18], with α = 0.05, power = 80%, and considering a drop-out rate of ∼10%, the final sample size was estimated to be 22 per group. Using Kolmogorov-Smirnov test the normal distribution of variables was evaluated. The paired t-test was used to analyze the data within groups at baseline and at the end of the study. Comparing effects of intervention on variables between the groups was measured using two-way ANOVA. Data were reported by means ± standard deviations (SD) and *P* <  0.05 was considered significant. Data were analyzed using SPSS version 24 (SPSS, Inc.)

## Results

### Characteristics of the subjects

**Fig. 1:**
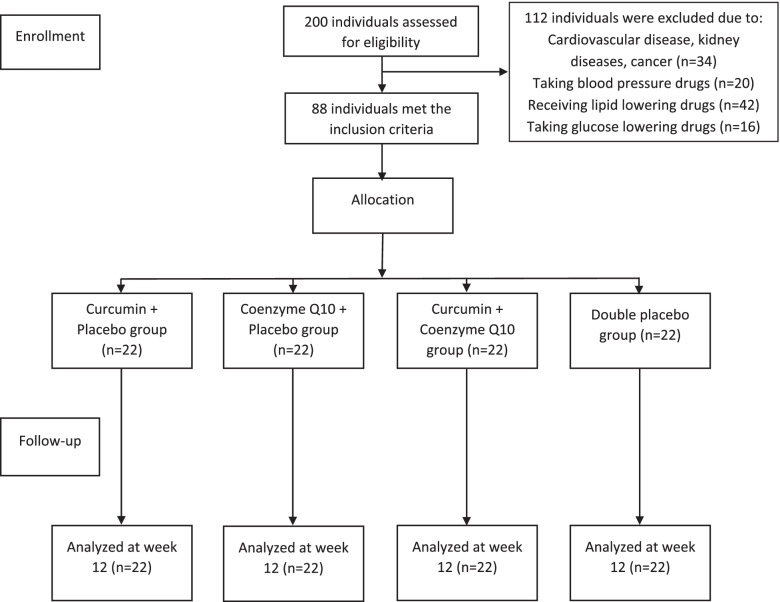
Eligibility, screening, and follow-up of subjects with MetS

After screening 200 subjects with MetS, 112 subjects did not meet the inclusion criteria due to having cardiovascular disease, kidney diseases or cancer (*n* = 34), taking blood pressure drugs (*n* = 20), receiving lipid lowering drugs (*n* = 42), and taking glucose lowering drugs (*n* = 16). Eighty-eight subjects were randomly assigned into four groups. All subjects completed the trial (Fig. 1). There were no significant differences between four groups in baseline characteristics (Table 1). There was no significant difference between four group in physical activity, intake of energy and intake of macronutrients during follow-up (*P* > 0.05) (Table 2). After the end of the study, no significant difference between the four groups in physical activity (*P* > 0.05) was found.

**Table 1:** Baseline characteristics of subjects with metabolic syndrome

	CP group (***n*** = 22)	QP group (***n*** = 22)	CQ group (***n*** = 22)	DP group (***n*** = 22)	***P***
**Age,** y	38.8 ± 4.9	39.0 ± 4.5	37.7 ± 5.0	39.5 ± 5.0	0.41
**Sex,** n (%)					0.19
Male	14 (64)	13 (59)	13 (59)	15 (68)	
Female	8 (36)	9 (41)	9 (41)	7 (32)	
**Physical activity,** MET-h/d	26.1 ± 2.6	27.8 ± 3.8	24.9 ± 4.1	28.1 ± 5.1	0.71
**Energy intake,** kcal/d	2285.7 ± 35	2275.3 ± 42	2290.1 ± 38	2210.8 ± 40	0.26
**TG**, mg/dL	224.7 ± 66.9	244.6 ± 95.2	236.8 ± 64.1	231.7 ± 80.7	0.58
**HDL-c**, mg/dL	32.0 ± 5.6	32.9 ± 4.7	31.2 ± 7.5	33.3 ± 8.5	0.41
**SBP**, mmHg	130.2 ± 7.5	127.2 ± 10.8	126.2 ± 10.4	135.1 ± 14.2	0.82
**DBP**, mmHg	91.0 ± 8.4	92.0 ± 7.9	93.1 ± 6.7	92.6 ± 6.5	0.84
**WC**, cm	101.0 ± 8.4	102.1 ± 6.5	102.7 ± 6.6	101.8 ± 7.7	0.13
**FPG**, mg/dL	130.1 ± 9.7	128.2 ± 14.3	121.2 ± 9.8	130.1 ± 14.7	0.27

Data are expressed as mean ± standard deviation. *CP* curcumin plus placebo, *QP* coenzyme Q10 plus placebo, *CQ* curcumin plus coenzyme Q10, *DP* double placebo, *MET-h* metabolic equivalent task hours, *TG* triglyceride, *HDL-c* high density lipoprotein-cholesterol, *SBP* systolic blood pressure, *DBP* diastolic blood pressure, *WC* waist circumference, *FPG* fasting plasma glucose

**Table 2:** Dietary intakes and physical activity in subjects with metabolic syndrome^a^

Variables	CP group (***n*** = 22)	QP group (***n*** = 22)	CQ group (***n*** = 22)	DP group (***n*** = 22)	***P***	
					***P***_***baseline***_	***P***_***between groups***_
**Energy**, Kcal/day					0.25	0.45
Week 0	2285.7 ± 35.5	2275.3 ± 42.2	2290.1 ± 38.4	2210.7 ± 40.8		
Week 12	2308.5 ± 38.2	2301.6 ± 30.3	2315.2 ± 31.3	2308.7 ± 40.3		
P_***within groups***_	0.56	0.60	0.67	0.85		
**Carbohydrate**, gr/day					0.46	0.31
Week 0	298.7 ± 4.5	290.3 ± 3.5	285.6 ± 6.5	295.8 ± 7.4		
Week 12	290.4 ± 5.8	286.1 ± 3.8	281.6 ± 4.8	289.1 ± 5.8		
P_***within groups***_	0.30	0.70	0.42	0.63		
**Fat**, gr/day					0.91	0.76
Week 0	70.2 ± 2.1	69.2 ± 3.2	73.2 ± 2.5	71.1 ± 4.1		
Week 12	69.2 ± 2.8	66.2 ± 1.9	71.1 ± 2.1	70.3 ± 4.5		
P_***within groups***_	0.30	0.09	0.15	0.45		
**Protein**, gr/day					0.41	0.32
Week 0	88.2 ± 5.3	87.4 ± 5.6	84.5 ± 3.4	85.2 ± 4.1		
Week 12	86.1 ± 4.3	85.1 ± 4.6	83.6 ± 3.8	84.4 ± 4.5		
P_***within groups***_	0.15	0.40	0.60	0.63		
**Physical activity,** MET-h/d					0.71	0.60
Week 0	26.1 ± 2.6	27.8 ± 3.8	24.9 ± 4.1	28.1 ± 5.1		
Week 12	25.8 ± 2.8	27.1 ± 3.4	25.1 ± 4.4	27.8 ± 5.5		
P_***within groups***_	0.41	0.60	0.15	0.24		

*CP* curcumin plus placebo, *QP* coenzyme Q10 plus placebo, *CQ* curcumin plus coenzyme Q10, *DP* double placebo, *MET-h* metabolic equivalent task hours

^a^Data are expressed as mean ± standard deviation (SD)

### Primary outcomes

After the end of the study, a significant reduction in term of TG in the CP (*P* <  0.001) and CQ (*P* = 0.01) groups, and a trend to improvement but not significant in the QP (*P* = 0.08) was found (Table 3). However, reduction of TG in the CP group compared to the three other groups was significantly greater (mean difference of CP: − 58.0 ± 49.1, QP: − 9.8 ± 30.4, CQ: − 16.0 ± 48.4, DP: 6.1 ± 59.5; *P* <  0.001) (Table 3). Furthermore, there was a significant improvement only in the CP group in HDL-c (mean difference of CP: 10.0 ± 5.3, QP: 1.0 ± 4.5, CQ: 1.8 ± 4.6, DP: 0.9 ± 6.9; *P* <  0.001) (Table 3). However, there was no significant difference between the four groups in terms of SBP (*P* = 0.72), DBP (*P* = 0.64), WC (*P* = 0.45) and FPG (*P* = 0.30) at the end of the study (Table 3). No serious adverse events were reported during follow-up.

**Table 3:** Effects of curcumin and/or coenzyme Q10 supplementation on metabolic syndrome components ^a^

Metabolic syndrome components	CP group (***n*** = 22)	QP group (***n*** = 22)	CQ group (***n*** = 22)	DP group (***n*** = 22)	***P***	
					***P***_***baseline***_	***P***_***between groups***_
**TG**, mg/dL					0.58	< 0.001
Week 0	224.7 ± 66.9	244.6 ± 95.2	236.8 ± 64.1	231.7 ± 80.7		
Week 12	166.7 ± 72.8	234.8 ± 39.4	220.8 ± 73.7	237.8 ± 88.6		
Mean difference	−58.0 ± 49.1	−9.8 ± 30.4	−16.0 ± 48.4	6.1 ± 59.5		
P_***within groups***_	< 0.001	0.08	0.01	0.51		
**HDL-c**, mg/dL					0.41	< 0.001
Week 0	32.0 ± 5.6	32.9 ± 4.7	31.2 ± 7.5	33.3 ± 8.5		
Week 12	42.0 ± 5.9	33.9 ± 4.6	33.0 ± 7.6	34.2 ± 8.6		
Mean difference	10.0 ± 5.3	1.0 ± 4.5	1.8 ± 4.6	0.9 ± 6.9		
P_***within groups***_	< 0.001	0.43	0.25	0.40		
**SBP**, mmHg					0.82	0.72
Week 0	130.2 ± 7.5	127.2 ± 10.8	126.2 ± 10.4	135.1 ± 14.2		
Week 12	130.8 ± 8.4	126.2 ± 9.9	125.2 ± 9.7	134.9 ± 15.2		
Mean difference	0.6 ± 8.1	−1.0 ± 9.7	−1.0 ± 9.3	−0.2 ± 12.8		
P_***within groups***_	0.74	0.42	0.40	0.64		
**DBP**, mmHg					0.84	0.64
Week 0	91.0 ± 8.4	92.0 ± 7.9	93.1 ± 6.7	92.6 ± 6.5		
Week 12	91.5 ± 6.6	91.1 ± 8.5	92.1 ± 4.3	91.8 ± 9.9		
Mean difference	0.5 ± 7.5	− 0.9 ± 8.3	−1.0 ± 5.5	− 0.8 ± 7.9		
P_***within groups***_	0.31	0.51	0.45	0.56		
**WC**, cm					0.33	0.45
Week 0	101.0 ± 8.4	102.1 ± 6.5	102.7 ± 6.6	101.8 ± 7.7		
Week 12	100.2 ± 8.5	101.5 ± 7.3	102.1 ± 8.6	101.4 ± 7.4		
Mean difference	−0.8 ± 4.0	− 0.6 ± 3.6	−0.6 ± 4.0	−0.4 ± 3.9		
P_***within groups***_	0.08	0.11	0.10	0.20		
**FPG**, mg/dL					0.27	0.30
Week 0	130.1 ± 9.7	128.2 ± 14.3	121.2 ± 9.8	130.1 ± 14.7		
Week 12	126.2 ± 8.5	126.2 ± 15.4	118.9 ± 8.2	126.3 ± 13.8		
Mean difference	−3.9 ± 7.3	−2.0 ± 9.3	−3.3 ± 7.3	−3.8 ± 9.7		
P_***within groups***_	0.40	0.67	0.45	0.40		

*CP* curcumin plus placebo, *QP* coenzyme Q10 plus placebo, *CQ* curcumin plus coenzyme Q10, *DP* double placebo, *TG* triglyceride, *HDL-c* high density lipoprotein-cholesterol, *SBP* systolic blood pressure, *DBP* diastolic blood pressure, *WC* waist circumference, *FPG* fasting plasma glucose

^a^Data are expressed as mean ± standard deviation (SD)

### Secondary outcomes

There was a significant reduction in term of TC in the CP group (*P* <  0.001), and a trend to reduction but not significant in QP group (*P* = 0.06) (Table 4). The CP group showed a greater reduction in TC compared to three other groups (mean difference of CP: − 30.9 ± 35.1, QP: − 10.1 ± 30.2, CQ: − 7.0 ± 36.3, DP: − 0.7 ± 43.4; *P* <  0.001) (Table 4). Moreover, there was a significant reduction in LDL-c (mean difference of CP: − 14.1 ± 6.2, QP: − 2.3 ± 8.3, CQ: − 1.0 ± 9.3, DP: − 2.0 ± 8.4; *P* <  0.001) in the CP group, but these variables in three other groups remained without significant improvement (Table 4). No significant difference between the four groups was found in terms of weight (*P* = 0.19) and BMI (*P* = 0.45) at the end of the study (Table 4).

**Table 4:** Effects of curcumin and/or coenzyme Q10 supplementation on lipid profile and body composition^a^

Variables	CP group (***n*** = 22)	QP group (***n*** = 22)	CQ group (***n*** = 22)	DP group (***n*** = 22)	***P***_***baseline***_	***P***_***between groups***_
**Lipid profile**^b^						
**TC,** mg/dL					0.52	< 0.001
Week 0	217.4 ± 37.4	238.4 ± 37.9	228.2 ± 47.9	238.4 ± 68.1		
Week 12	186.5 ± 30.4	228.3 ± 39.8	221.2 ± 40.7	237.7 ± 53.9		
Mean difference	−30.9 ± 35.1	−10.1 ± 30.2	−7.0 ± 36.3	−0.7 ± 43.4		
P_***within groups***_	< 0.001	0.06	0.15	0.70		
**LDL-c,** mg/dL					0.46	< 0.001
Week 0	89.4 ± 6.5	90.8 ± 8.6	96.3 ± 11.2	89.3 ± 10.1		
Week 12	75.3 ± 5.4	88.5 ± 8.4	95.3 ± 11.4	87.3 ± 7.4		
Mean difference	−14.1 ± 6.2	−2.3 ± 8.3	−1.0 ± 9.3	−2.0 ± 8.4		
P_***within groups***_	< 0.001	0.35	0.42	0.40		
**Body composition indices**^c^						
**Weight,** Kg					0.07	0.19
Week 0	92.1 ± 8.2	94.0 ± 10.1	91.0 ± 10.8	92.1 ± 10.5		
Week 12	90.1 ± 8.1	92.2 ± 15.8	89.1 ± 10.2	91.1 ± 10.1		
Mean difference	−2.0 ± 7.6	−1.8 ± 12.6	−1.9 ± 9.7	−1.0 ± 9.5		
P_***within groups***_	0.07	0.09	0.08	0.15		
**BMI,** Kg/m^2^					0.21	0.45
Week 0	30.0 ± 4.6	30.7 ± 4.9	29.7 ± 5.1	30.0 ± 4.7		
Week 12	29.4 ± 4.2	30.1 ± 5.6	29.1 ± 5.0	29.7 ± 4.7		
Mean difference	−0.6 ± 3.9	−0.6 ± 4.5	−0.6 ± 4.5	−0.3 ± 4.4		
P_***within groups***_	0.34	0.41	0.40	0.45		

*CP* curcumin plus placebo, *QP* coenzyme Q10 plus placebo, *CQ* curcumin plus coenzyme Q10, *DP* double placebo, *TC* total cholesterol, *LDL-c* low density lipoprotein-cholesterol, *BMI* body mass index, *TG* triglyceride, *HDL-c* high density lipoprotein-cholesterol, *WC* waist circumference

^a^Data are expressed as mean ± standard deviation (SD). ^b^Values of TG and HDL-c, which are parameters of lipid profile, are expressed in Table 2. ^c^Values of WC, which is a variable of body composition, is expressed in Table 2

## Discussion

To our knowledge the present study was the first randomized clinical trial (RCT) investigating the effects of curcumin and/or coenzyme Q10 supplementation in subjects with MetS. We found that curcumin supplementation improves lipid profile, but has no effect on body composition, hypertension and FPG. On the other hand, supplementation with coenzyme Q10 as well as curcumin plus coenzyme Q10 showed no significant effects on lipid profile, body composition, hypertension and FPG.

The evidence from in vitro and in vivo studies revealed the mechanisms that curcumin improves dyslipidemia [16, 34]. Curcumin downregulates key factors in the lipogenesis like 3-hydroxy-3-methylglutaryl-CoA (HMG-CoA) reductase, sterol regulatory element-binding proteins (SREBPs) and fatty acid synthase, and stimulates lipid excretion as well as mobilization from adipose tissue [16, 34]. Consistent with the results of the present study, an improvement in lipid profile of subjects with MetS after 8-week curcumin supplementation (1000 mg/day) was found in the trial of Panahi et al. [18]. In addition, the study of Adab et al. [35] confirmed that curcumin intake (2100 mg/d powdered of turmeric) for 8 weeks reduces TG, TC and LDL-c in patients with T2DM. However, study of Baum et al. [36] showed that curcumin supplementation (1000 mg/day) for 6 months has no effect on lipid profile in healthy adults. The differences in health status of subjects can be a logical reason for this contrast. Our finding did not show any significant improvement in lipid profile after supplementation with coenzyme Q10. In line with our findings, in the study of Raygan et al. [37], no significant improvement in lipid profile was observed after coenzyme Q10 supplementation for 12 weeks among subjects with MetS. In addition, Gholnari et al. [25] found that 12-week supplementation with coenzyme Q10 has no effect on lipid profile in patients with diabetic nephropathy. In contrast to our study, Zhang et al. [24] found that treatment with coenzyme Q10 (120 mg/day) for 24 weeks improves lipid profile in dyslipidemic individuals. In addition, Derosa et al. [38] demonstrated that 3-month CoQ10 supplementation (100 mg/day) decreases TC and LDL-c in dyslipidemic subjects. It seems, longer duration interventions with coenzyme Q10 has a beneficial effect in dyslipidemic subjects, but, it has no effect in diabetic or MetS subjects.

Based on our findings, although the CQ group (curcumin plus coenzyme Q10) showed some effects on dyslipidemia, the CP group (curcumin plus placebo) had greater effects. In contrast to similarity in some lipid-lowering mechanisms of curcumin and coenzyme Q10 [19, 20, 34], it seems coenzyme Q10 neutralized the therapeutic effects of curcumin on dyslipidemia.

Our findings demonstrated that curcumin and/or coenzyme Q10 supplementation have no effect on hypertension. Contrary to our study, previous evidence suggested that curcumin by mechanisms like increasing nitric oxide bioavailability and reducing oxidative stress as well as endothelial dysfunction and ameliorating aortic stiffening can improve hypertension [39]. A meta-analysis on RCTs suggested that consuming curcumin/turmeric has no effect on blood pressure and may improve SBP in long duration interventions [17]. On the other hand, studies suggested that coenzyme Q10 by inducing vasodilatation via impact on the endothelium and peripheral vascular resistance, as well as increasing nitric oxide levels and reducing lipid peroxidation, improves hypertension. In contrast with our results, Hodgson et al. [30] found that coenzyme Q10 (200 mg/day) for 12 weeks can reduce blood pressure in patients with T2DM. In line with our results, study of Young et al. [22] showed that coenzyme Q10 supplementation (100 mg/day) for 12 weeks cannot reduce blood pressure in hypertensive subjects with MetS. Furthermore, in the study of Eriksson et al. [26], blood pressure remained unchanged after 6-month coenzyme Q10 supplementation (100 mg/day). Higher doses of coenzyme Q10 probably has a beneficial effect on hypertension.

We found that after supplementation with curcumin and/or coenzyme Q10, anthropometric variables did not change significantly. In contrast to our finding, previous studies showed that curcumin can reduce weight and WC by increasing the metabolic rate, downregulating adipocyte transcriptional factors like peroxisome proliferator-activated receptor γ (PPAR-γ) and inhibiting preadipocyte differentiation [40–42]. In line with our results, the study of Saraf-bank et al. [43] suggested that curcumin supplementation (500 mg/day) for 10 weeks has no beneficial effect on weight in subjects with obesity. In addition, the study of Adab et al. [35] showed no significant reduction in weight in patients with T2DM after curcumin supplementation (2100 mg powdered of turmeric). Longer duration of curcumin supplementation may be useful to improve body composition. Some previous evidence showed coenzyme Q10 may inhibit adipocyte differentiation by mediating AMP-activated protein kinase (AMPK) and peroxisome proliferator-activated receptor α (PPAR-α) pathways, as well as decrease gene expression of enzymes involved in the lipid synthesis such as fatty acid synthase and Acetyl-CoA carboxylase [44, 45]. In line with our study, Eriksson et al. [26] demonstrated that coenzyme Q10 intake (100 mg/day) for 6 months has no effect on BMI in patients with T2DM. Furthermore, Izadi et al. [46] reported that coenzyme Q10 (200 mg/day) compared to the placebo has no significant effect on visceral adiposity in women with polycystic ovary syndrome (PCOS).

Finally, our study showed there is no significant improvement in FPG after 12-week curcumin and/or coenzyme Q10 supplementation. Some evidence reported that curcumin increases insulin secretion from pancreatic cells and regulates postprandial insulin responses, reduces NF-κB activity, increases activity of AMPK and anti-oxidant transcription factors like Foxo1, thereby can improve glycemic control [47–49]. Consistent with our study, in the study of Thota et al. [29] no significant improvement in glycemic control parameters such as FPG and HbA1c was found after supplementation with curcumin (180 mg/day) for 12 weeks among subjects with T2DM; however, curcumin significantly improved insulin and homeostatic model assessment for insulin resistance (HOMA-IR). In contrast to our findings, NA et al. [50] suggested that curcumin supplementation (300 mg/day) reduces FPG, HbA1c and HOMA-IR in patients with T2DM. On the other hand, studies suggested that coenzyme Q10 by modulating receptors of insulin and adiponectin, and regulating tyrosine kinase (TK), phosphatidylinositol kinase (PI3K), and glucose transporters improves glycemic control [21]. In line with our results, in the study of Gholnari et al. [25], coenzyme Q10 supplementation (100 mg/day) for 12 weeks did not show improvement in FPG in patients with diabetic nephropathy; However, in this study insulin and HOMA-IR improved significantly. In addition, Eriksson et al. [26] found that coenzyme Q10 supplementation (100 mg/day) for 6 months did not change glycemic control. Moreover, the study of Lee et al. [51] showed that 12-week coenzyme Q10 supplementation (200 mg/day) could not improve glycemic control in obese subjects. Based on literature, it seems higher doses of curcumin as well as coenzyme Q10 have a greater effect on insulin and HOMA-IR than FPG.

Our study has some strengths such as assessing the effect of curcumin along with coenzyme Q10 in a separate group, and the high rate of compliance (100%). However, our study has some limitations. We did not evaluate the dietary intake of curcumin and coenzyme Q10. In addition, we did not measure levels of insulin and HOMA-IR, which could help to accurate conclusion in the field of glycemic control. Moreover, subjects were enrolled sequentially (rather than all at once), which could lead to selection bias.

## Conclusions

In conclusion, our finding indicated that curcumin supplementation (especially by its effects on dyslipidemia) is more effective than coenzyme Q10 as well as the combination of curcumin and coenzyme Q10 in the management of MetS. However, curcumin, coenzyme Q10 and their combination have no effect on body composition, hypertension and glycemic control.

## Data Availability

The data and materials of the current study is available from the corresponding author on reasonable request.
